# Machine learning for early prediction of sepsis-associated acute brain injury

**DOI:** 10.3389/fmed.2022.962027

**Published:** 2022-10-03

**Authors:** Chenglong Ge, Fuxing Deng, Wei Chen, Zhiwen Ye, Lina Zhang, Yuhang Ai, Yu Zou, Qianyi Peng

**Affiliations:** ^1^Department of Critical Care Medicine, Xiangya Hospital, Central South University, Changsha, China; ^2^National Clinical Research Center for Geriatric Disorders, Changsha, China; ^3^Hunan Provincial Clinical Research Center for Critical Care Medicine, Changsha, China; ^4^Department of Oncology, Xiangya Hospital, Central South University, Changsha, China; ^5^Department of Anesthesia, Xiangya Hospital, Central South University, Changsha, China

**Keywords:** sepsis-associated encephalopathy, machine learning, prediction, MIMIC III, light gradient boosting machine

## Abstract

**Background:**

Sepsis-associated encephalopathy (SAE) is defined as diffuse brain dysfunction associated with sepsis and leads to a high mortality rate. We aimed to develop and validate an optimal machine-learning model based on clinical features for early predicting sepsis-associated acute brain injury.

**Methods:**

We analyzed adult patients with sepsis from the Medical Information Mart for Intensive Care (MIMIC III) clinical database. Candidate models were trained using random forest, support vector machine (SVM), decision tree classifier, gradients boosting machine (GBM), multiple layer perception (MLP), extreme gradient boosting (XGBoost), light gradients boosting machine (LGBM) and a conventional logistic regression model. These methods were applied to develop and validate the optimal model based on its accuracy and area under curve (AUC).

**Results:**

In total, 12,460 patients with sepsis met inclusion criteria, and 6,284 (50.4%) patients suffered from sepsis-associated acute brain injury. Compared other models, the LGBM model achieved the best performance. The AUC for both train set and test set indicated excellent validity (Trainset AUC 0.91, Testset AUC 0.87). Feature importance analysis showed that glucose, age, mean arterial pressure, heart rate, hemoglobin, and length of ICU stay were the top 6 important clinical factors to predict occurrence of sepsis-associated acute brain injury.

**Conclusion:**

Almost half of patients admitted to ICU with sepsis had sepsis-associated acute brain injury. The LGBM model better identify patients with sepsis-associated acute brain injury than did other machine-learning models. Glucose, age, and mean arterial pressure were the three most important clinical factors to predict occurrence of sepsis-associated acute brain injury.

## Introduction

Sepsis-associated encephalopathy (SAE) is defined by diffuse cerebral dysfunction, which is associated with sepsis in the absence of direct central nervous system (CNS) infection, structural abnormality or other types of encephalopathy (1). Patients with SAE have a higher intensive care unit (ICU) mortality than those suffering from sepsis alone (2–4). SAE might dramatically be aggravated by metabolic disturbances, use of antibiotics with significant neurotoxicity, and complications such as acute renal failure and hypoglycemia (5–7). In the clinic surroundings, SAE is diagnosed cognitive and neuropsychiatric disorders that documented by medical staff trained to identification, as well as a Glasgow coma score (GCS) < 15 or manifestations of delirium (including inattention, disorientation, altered thinking, decreased psychomotor activity and so on), and need to exclude sedative-related cognitive effects, primary CNS disease, metabolic encephalopathy, and toxicosis (8). Due to the lack of an early diagnosis system, diagnosis and management of SAE are often delayed, leading to significant morbidity and mortality. Early diagnosis and treatment for brain injury are crucial for the survival and prognosis of sepsis patients.

There are only limited data on the prediction for septic patients developing SAE. Some risk scores and biomarkers, including nomogram, calcium-binding protein A8 (S100A8), tumor necrosis factor receptor-associated factor 6 (TRAF6), and S100B, were recently used to predict SAE (9–11). However, these biomarkers are not always applicable in clinical practice, and these methods lacks both sensitivity and specificity for prediction of SAE. Therefore, new predictors such as clinical indicators are needed to assist in prediction.

It is generally accepted that machine learning is helpful for the early surveillance and identification of sepsis (9, 12, 13). Although nomograms have been used for the prediction of SAE, the sensitivity and specificity are relatively low (9, 14). Few interpretable machine-learning methods have been used for clinical practice of SAE. Therefore, an understanding algorithm are crucial to enhance the sensitivity of prediction. Machine learning is an excellent mathematical model for solving the complex relationship between diseases and potential risk factors because it can learn from sample data, instead of assuming a global relationship between them based on all samples by human experts. This study aims to develop machine-learning (ML) models for early prediction of sepsis-associated acute brain injury. The best performing model was selected for further prediction.

## Materials and methods

### Study design

In this large dataset, the final ML model was developed in three steps. First, we developed the models using a conventional logistic regression and seven machine-learning methods. Second, the evaluation of model performance in a validation cohort were compared. Finally, the best-fitting model was selected and constructed.

### Source of data

The data of this retrospective study came from MIMIC-III, an openly available US-based critical care database. The description of MIMIC-III can be found in the literature (15). The MIMIC III database includes clinical information relating to patients admitted to the ICUs of Beth Israel Deaconess Medical Center (single) in Boston from 2001 to 2016, which is approved by the Massachusetts Institute of Technology Institutional Review Boards. Patients were selected using the PostgreSQL 9.6 software from the latest version (MIMIC-III v1.4), which was released on 2 September 2016. After successfully completing the Collaborative Institutional Training Initiative Program course (Record ID 35897056), we were allowed to utilize the data from MIMIC-III.

### Participants and data extraction

Structure query language (SQL) was used to extract data from the MIMIC III database by PgAdmin (version 4.1, Bedford, USA). The study inclusion criteria included: (1) If a patient had multiple ICU admissions, only the first admission was included, (2) patients with a diagnosis of Sepsis (Sepsis-3) based on the code and method established by Angus et al. (16) and the Sequential Organ Failure Assessment (SOFA) score ≥ 2. Exclusion criteria included the following: (1) age < 18 years; (2) without an evaluation of Glasgow Coma Scale (GCS); (3) length of ICU stay > 100 days.

For the final study, the data on the first day of ICU admission were collected from the MIMIC III database: including age, gender, weight, comorbidity, mean value of vital signs, baseline laboratory data (the first measurement on the first day), SOFA score, simplified acute physiology score II (SAPSII), use of vasopressors, renal replacement therapy (RRT), mechanical ventilation, infection sites, microorganisms, length of ICU stay, length of hospital stay.

### Sepsis-associated acute brain injury

According to the previous literature reports, sepsis-associated acute brain injury were defined as sepsis accompanying GCS score ≤ 14 at ICU admission or subacute delirium (icd9_code = 2,931) or delirium due to conditions classified elsewhere (icd9_code = 2,930) (9, 17, 18). The delirium caused by dementia, alcohol or drug abuse, mental illness, and other primary neurological disorders are excluded. For sedated and postoperative patients, GCS score were extracted before any administration of sedative drug or surgery. Those patients had to be excluded as well: (1) Primary brain injury (subarachnoid hemorrhage, intracerebral hemorrhage, traumatic brain injury, cerebral infarction, epilepsy, or intracranial infection); (2) dementia; (3) depression; (4) psychoses;(5) alcohol or drug abuse; (6) hypertensive encephalopathy, metabolic encephalopathy, hepatic encephalopathy, and other disease affecting consciousness; (7) severe acid-base disturbance or electrolyte imbalance, including PaCO_2_ > 80 mmHg, hyponatremia (<120 mmol/l), hyperglycemia (>180 mg/dl), or hypoglycemia (<54 mg/dl).

### Model development

**FIGURE 1:**
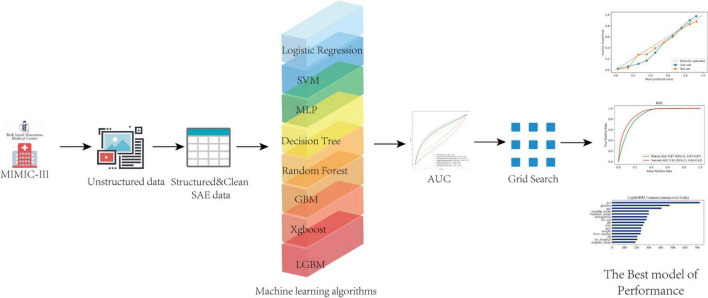
A flow chart of the study. AUC, area under the curve, SVC, support vector classification; MLP, multi-layer perceptron; XGB, extreme gradient boosting; LGBM, light gradient boosting machine.

The dataset was split into two groups to develop the models a training set with 70% of the individuals and a test set of the size of 30%. Predictive models-based machine learning were built with (1) logistic regression (2) support vector machine (SVM); (3) decision tree classifier; (4) random forest; (5) gradients boosting machine (GBM); (6) multiple layer perception (MLP); (7) extreme gradient boosting (XGBoost); (8) light gradients boosting (LGBM). SVM and logistic regression are classic machine learning algorithms, which play a key role in formulating the base line for the establishment of the model. SVM is a binary classification technique that uses the training dataset to predict an optimal hyperplane in an n-dimensional space (19). Logistic regression can describe a relationship between the categorical variable with one or more nominal, ordinal, interval variables, which has a wide range of applications in traditional medical statistics. MLP is a simple neural network with some layers network of hidden neurons, whose parameters we chosen is “solver = “lbfgs,” max_iter = 100.” Among them, decision tree, random forest, GBM, XGBoost and LGBM are the class tree models using ensemble learning idea. Ensemble learning is a general meta-approach to machine learning that seeks better predictive performance by combining the predictions from multiple models. The LGBM and XGBoost are both asymmetric trees and developed methods recently, but LGBM grows leaf-wise while Xgboost grows level-wise (20, 21). Firstly, in the model-comparison phase, we tested and compared the performances of the seven predictive models by the area under curves (AUCs) of the receiver operating characteristic curves (ROC). The cross-validation procedure was repeated five times (fivefold cross-validation). Then, we selected the model that achieved the highest AUC value for further optimizing the parameters using the grid tuning method. The classification results of the final model are then shown as a confusion matrix. With the test set combined with the best model, we formed calibration curves, and the accuracy of the calibration validated. Finally, all features used by the model were ranked by measured Gini impurity. The machine learning-based classifiers are implemented using python version 3.6 library “Sklearn,” “xgboost,” “lightgbm.” The workflow (Figure 1) code about development models is available and open source (MIT license) on Github.^1^

### Statistical analysis

Continuous variables are presented as median with interquartile range (IQR) due to their non-normal distribution. Categorical variables are presented as frequency and percentage. Differences of continuous variables between independent groups have been analyzed using Mann-Whitney *U*-test. The Chi-square test was used to compare categorical variables between groups.

Missing values for all screening variables were less than 5%. Single imputation (Simple Linear Regression) was used for these variables with missing values, which included mean temperature, mean SpO_2_, platelet, hemoglobin, glucose, potassium, sodium, and creatinine (Supplementary Table 1). Statistical analysis was carried out using software Stata 15.1^2^ and R 4.0.0^3^ for the Windows operative system. *P*-values < 0.05 were considered statistically significant.

## Results

### Participants and baseline characteristics

**FIGURE 2:**
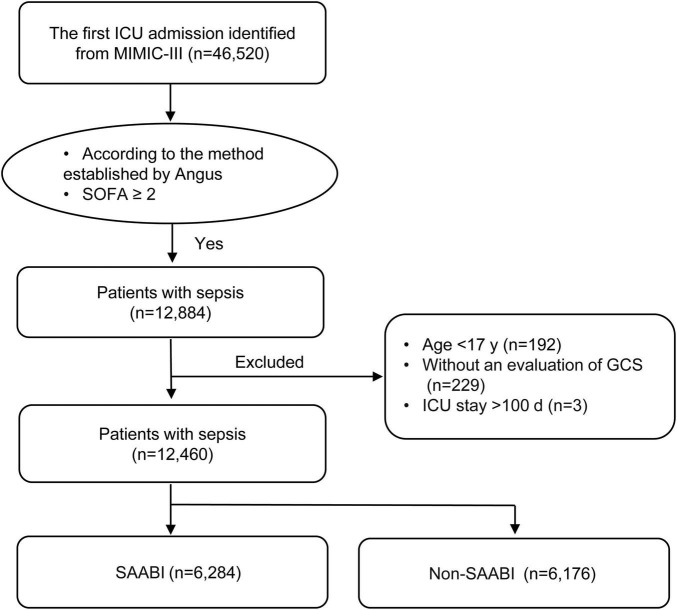
Flowchart of patient screening and selection. ICU, intensive care unit; MIMIC-III, Medical Information Mart for Intensive Care III; SOFA, sequential organ failure assessment; GCS, Glasgow Coma Scale; SAABI, sepsis-associated acute brain injury.

Of 46,520 critically ill patients with the first ICU admission obtained from the MIMIC-III database, 12,884 with sepsis were included. Of these, 424 were excluded according to the exclusion criteria. Finally, a total of 12,460 individuals were included into the analysis, and sepsis-associated acute brain injury was observed in 6,284 (50.4%) patients (Figure 2).

Characteristics at baseline of all participants were described in Table 1. The median age was 72 years (IQR, 59–82 years), and 3,218 of 6,284 patients (51.2%) were male in sepsis-associated acute brain injury. Acute kidney injury (AKI) was the most common (4,554 of 6,284, 72.5%) comorbidities, followed by cardiovascular diseases (4,048 of 6,284, 64.4%) and hypertension (3,256 of 6,284, 51.8%) in SAE. Patients with sepsis-associated acute brain injury at admission were more critically ill indicated by higher SOFA score [6 (IQR 4–8) vs. 4 (IQR 3–6), *p* < 0.001] and SAPS II score [43 (IQR 34–53) vs. 37 (IQR 29–45), *p* < 0.001], had a higher proportion of medical treatments, such as vasopressor use (50.4 vs. 36.1%, *P* < 0.001) and ventilation use (66.2 vs. 44.9%, *P* < 0.001), and had longer ICU stay time, than those who did not. Compared with non- sepsis-associated acute brain injury patients, patients with sepsis-associated acute brain injury were more likely to suffer from fungal infection (10.5 vs. 7.5%, *P* < 0.001).

**TABLE 1:** Baseline characteristics of patients at ICU admission.

Variables	All patients	Non-SAABI	SAABI	*P*-value
			
	*n* = 12,460	*n* = 6,176	*n* = 6,284	
**Demographics**				
Male, *n* (%)	6,559 (52.6)	3,341 (54.1)	3,218 (51.2)	0.001
Age (y), median [Q1, Q3]	69 [56, 80]	66 [53, 78]	72 [59, 82]	< 0.001
Weight (kg), median [Q1, Q3]	77 [65, 91]	77 [66, 93]	76 [63, 90]	< 0.001
**Comorbidity, *n* (%)**				
Cardiovascular diseases	7,544 (60.5)	3,496 (56.6)	4,048 (64.4)	< 0.001
Peripheral vascular diseases	1,055 (8.5)	511 (8.3)	544 (8.7)	0.462
Hypertension	6,524 (52.4)	3,268 (52.9)	3,256 (51.8)	0.226
Chronic pulmonary diseases	2,975 (23.9)	1,494 (24.2)	1,481 (23.6)	0.427
Diabetes	869 (7.0)	448 (7.3)	421 (6.7)	0.238
AKI	8,344 (67.0)	3,790 (61.4)	4,554 (72.5)	< 0.001
Liver disease	1,743 (14.0)	1,114 (18.0)	629 (10.0)	< 0.001
ARDS	100 (0.8)	71 (1.1)	29 (0.5)	< 0.001
Coagulopathy	2,215 (17.8)	1,190 (19.3)	1,025 (16.3)	< 0.001
Obesity	687 (5.5)	345 (5.6)	342 (5.4)	0.755
Anemia	712 (5.7)	382 (6.2)	330 (5.3)	0.027
History of TBI	11 (0.1)	5 (0.1)	6 (0.1)	1
History of stroke	261 (2.1)	133 (2.2)	128 (2.0)	0.695
Other neurological diseases	1,667 (13.4)	842 (13.6)	825 (13.1)	0.423
**Severe score, median [Q1, Q3]**				
SOFA	5 [3, 7]	4 [3, 6]	6 [4, 8]	< 0.001
SAPSII	40 [31, 49]	37 [29, 45]	43 [34, 53]	< 0.001
**Vital signs, median [Q1, Q3]**				
Mean heartrate (min^–1^)	87 [76, 99]	86 [75, 99]	87 [76, 99]	0.011
Mean arterial pressure (mmHg)	75 [68, 82]	75 [69, 83]	74 [68, 81]	< 0.001
Mean respiratory rate (min^–1^)	19 [16, 22]	19 [17, 22]	19 [16, 22]	< 0.001
Mean temperature (°C)	36 [36, 37]	36 [36, 37]	36 [36, 37]	0.047
Mean SpO_2_ (%)	97 [95, 98]	97 [95, 98]	97 [96, 98]	< 0.001
**Laboratory tests, median [Q1, Q3]**				
WBC (K/μl)	11.3 [7.9, 15.8]	10.9 [7.6, 15.4]	11.7 [8.2, 16.2]	< 0.001
Platelet (K/μl)	197 [135, 273]	195 [129, 268]	198 [140, 278]	< 0.001
Hemoglobin (g/dl)	10.6 [9.3, 12.2]	10.7 [9.4, 12.4]	10.5 [9.3, 12]	< 0.001
Glucose (mg/dl)	126 [104, 160]	123 [102, 156]	128 [106, 163]	< 0.001
Sodium (mmol/l)	139 [136, 142]	138 [135, 141]	139 [136, 142]	< 0.001
Creatinine (K/μl)	1.2 [0.8, 1.8]	1.1 [0.7, 1.7]	1.2 [0.7, 1.9]	0.448
Bilirubin (EU/dl)	0.7 [0.4, 1.6]	0.7 [0.3, 1.5]	0.8 [0.4, 1.7]	0.001
Lactate (mmol/l)	1.9 [1.3, 2.5]	1.9 [1.4, 2.5]	1.9 [1.3, 2.6]	0.106
PO_2_ (mmHg)	154 [91, 205]	154 [93, 198]	154 [90, 218]	0.01
PCO_2_ (mmHg)	41 [36, 47]	41 [36, 46]	41 [36, 48]	< 0.001
PH	7.36 [7.33, 7.40]	7.36 [7.34, 7.40]	7.36 [7.31, 7.41]	<0.001
Metabolic acidosis, *n* (%)	461 (3.7)	183 (3)	278 (4.4)	< 0.001
**Medical treatments, *n* (%)**				
RRT	622 (5.0)	297 (4.8)	325 (5.2)	0.374
Vasopressor	5,392 (43.3)	2,228 (36.1)	3,164 (50.4)	< 0.001
Ventilation	6,936 (55.7)	2,773 (44.9)	4,163 (66.2)	< 0.001
**Infection site, *n* (%)**				
Intestinal infection	909 (7.3)	468 (7.6)	441 (7.0)	0.243
Urinary infection	4,426 (35.5)	2,166 (35.1)	2,260 (36.0)	0.306
Lung infection	4,481 (36.0)	2,163 (35.0)	2,318 (36.9)	0.032
Catheter related	1,042 (8.4)	507 (8.2)	535 (8.5)	0.561
Skin soft tissue	1,504 (12.1)	731 (11.8)	773 (12.3)	0.442
Abdominal cavity	1,462 (11.7)	683 (11.1)	779 (12.4)	0.022
**Microorganisms, *n* (%)**				
Gram-positive	2,986 (24.0)	1,404 (22.7)	1,582 (25.2)	0.002
Gram-negative	2,080 (16.7)	987 (16.0)	1,093 (17.4)	0.037
Fungus	1,124 (9.0)	464 (7.5)	660 (10.5)	< 0.001
Virus	47 (0.4)	15 (0.2)	32 (0.5)	0.023
Other microorganisms	191 (1.5)	98 (1.6)	93 (1.5)	0.68
**Length of stay, median [Q1, Q3]**				
LOS in ICU (days)	3.2 [1.8, 7.7]	2.9 [1.6, 6.4]	3.80 [1.9, 8.7]	< 0.001
LOS in hospital (days)	10.6 [6.0, 18.8]	10.0 [5.9, 17.7]	11.20 [6.3, 19.7]	< 0.001
**Mortality, *n* (%)**				
28-day mortality	2,623 (21.1)	1,106 (17.9)	1,517 (24.1)	< 0.001
ICU mortality	1,710 (13.7)	659 (10.7)	1,051 (16.7)	< 0.001
Hospital mortality	2,293 (18.4)	944 (15.3)	1,349 (21.5)	< 0.001

Non-parametric continuous data are presented as median (interquartile ranges), whereas categorical data are presented as frequency (percentage). SAABI, sepsis-associated acute brain injury; AKI, acute kidney injury; CKD, chronic kidney disease; ARDS, acute respiratory distress syndrome; TBI, traumatic brain injury; WBC, white blood cell; IQR, interquartile range; SOFA, sequential organ failure assessment; SAPSII, simplified acute physiology score; RRT, renal replacement therapy; LOS, length of stay.

### Model development and validation

A total of 52 clinical variables collected during the first 24 h after ICU admission. We have constructed a conventional logistic regression and seven machine learning binary classifiers in predicting the risk of the sepsis-associated acute brain injury: SVM, Decision Tree, Random Forest, GBM, MLP, Xgboost, LGBM, most of which are tree-like models that can filter the features by themselves using the conception of ensemble learning. Figure (AUC) depicts the performance of these predictive models and shows that the LGBM (AUC: 0.78) model can provide relatively better model fitting performance compared to other ML models (AUC in train set: Logistic Regression: 0.74, SVM: 0.72, Decision Tree: 0.71, RF: 0.75, GBM: 0.77, MLP: 0.76, Xgboost: 0.77). After adjusting and optimizing the parameters of the LGBM algorithm through grid search, the calibration curve for the LGBM model showed that the predicted risk is in good agreement with the actual risk. The predicted value of the model is close to the actual probability of the outcome. Therefore, LGBM was selected for further prediction in this study.

### Model performance

**FIGURE 3:**
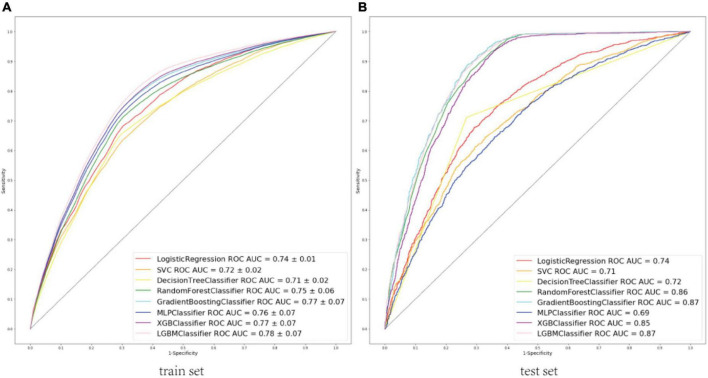
**(A)** ROCs of eight machine learning models to predict sepsis-associated acute brain injury in train set. **(B)** ROCs of eight machine learning models to predict sepsis-associated acute brain injury in the test set. ROC, Receiver operator characteristic curves; AUC, area under the curve; SVC, support vector classification; MLP, multi-layer perceptron; XGB, extreme gradient boosting; LGBM, light gradient boosting machine.

**FIGURE 4:**
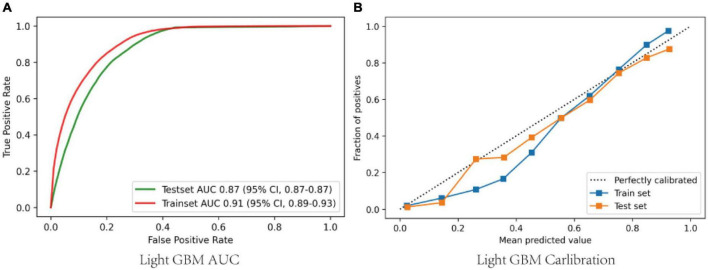
**(A)** ROCs of the LGBM after adjusting and optimizing the parameters; **(B)** Calibration curves of the LGBM after adjusting and optimizing the parameters. ROC, Receiver operator characteristic curves; GBM, gradient boosting machine.

The area under receiver operating characteristic curve (AU-ROC) was used to evaluate the model performance. The LGBM had a significantly greater area under the ROC curve (AUC) than other models (Figure 3). Figure 4 describes the AU-ROC and calibration for the LGBM model. The AUC for both trainset and testset indicated excellent validity (Figure 4A: Trainset AUC 0.91, Testset AUC 0.87). The calibration plot shows good agreement between the observed and predicted values (Figure 4B).

### Model explanation

**FIGURE 5:**
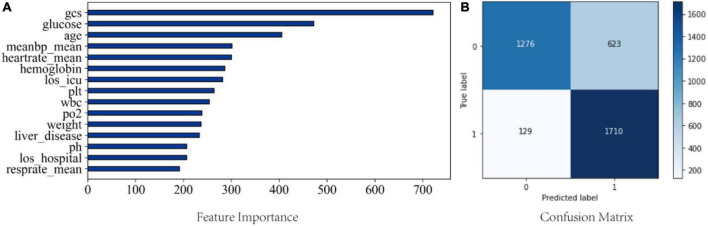
**(A)** Feature importance from the LGBM model; **(B)** Confusion matrix from the LGBM model. meanbp_mean, mean arterial pressure is presented as mean daily values; heartrate_mean, mean heart rate; los_icu, length of stay in ICU; plt, platelet; wbc, white blood cell; los_hosiptal, length of saty in hospital; resprate_mean, mean respiratory rate.

Feature importance was calculated using Gini impurity method for LGBM, which had the best performing ability in the validation cohort. Figure 5A shows the top 15 clinical features based on the mean Gini impurity value. Notably, besides GCS score, the features specific to sepsis-associated acute brain injury included glucose, age, and mean arterial pressure (MAP). Additionally, heart rate, hemoglobin, and length of ICU stay also supported a prediction of sepsis-associated acute brain injury. Figure 5B shows the confusion matrix.

## Discussion

In this retrospective analysis of a large critical care database, we found that 50.4% of sepsis-associated acute brain injury were identified at admission to the ICU. The 28-day mortality of sepsis-associated acute brain injury was 24.1%. Seven ML models and a conventional logistic regression model have been created and validated, according to 52 baseline variables included in the first 24 h after ICU admission, to predict the occurrence of SAE admitted to ICU. The LGBM model showed the best performance (Figure 4A: Trainset AUC 0.91, Testset AUC 0.87). Feature importance analysis of the LGBM model suggested that besides the GCS score, glucose, age, MAP, heart rate, hemoglobin, and length of ICU stay were the top 6 features, with the strongest impact on the prediction of sepsis-associated acute brain injury.

In recent years, machine learning-based methods are widely used in predicting sepsis-associated diseases. For instance, Layeghian Javan et al. (13) reported that machine learning techniques, especially ensemble algorithms have high potentials to be used in prognostic systems for sepsis-associated cardiac arrest. Zhao et al. (22) developed Categorical Boosting (CatBoost) model which was able to dynamically predict the risk of sepsis-induced coagulopathy (*SIC*) in septic patients better than conventional Logistic Regression and *SIC* scores. Reports on the prediction of SAE are relatively rare in the literature. Yang et al. (9) developed a nomogram method to predict 30-day mortality of patients with SAE, and found that the nomogram showed better discrimination with AU-ROC of 0.763 and 0.753 in the training and validation sets, respectively. Zhao et al. (14) concluded that predictors of SAE included age, quick sequential organ failure assessment (qSOFA), and the use of drugs including antibiotics, steroids, sedative medication, H2-antagonist, and heparin sodium injection by individualized prediction nomograms. The AUC was 0.743. However, it should be noted that the AUC was not sufficiently high, and the AUC value did not exceed 0.75. In this study, we demonstrated that ML methods were more accurate in predicting sepsis-associated acute brain injury than nomograms among patients with sepsis (Figure 4A: Trainset AUC 0.91, Testset AUC 0.87). Meanwhile, we compared various conventional ML and deep learning models. The result showed that the LGBM model could effectively enhance the prediction of sepsis-associated acute brain injury.

In our study, the importance of variables showed that glucose, age, MAP, heart rate, hemoglobin, and length of ICU stay were the most important risk factors that contribute to the predicted occurrence of sepsis-associated acute brain injury. In patients with severe sepsis, approximately 40% of them have baseline hyperglycemia and glycemic control improves patient outcomes (23). Recently it has been found that glycemic control with insulin attenuates SAE by inhibiting glial activation in septic rats (24). However, hypoglycemia is also independently associated with SAE (18). Thus, to prevent the occurrence of SAE, glycemic control should be more aggressive in the initial stage of sepsis. Our study also found that the age was closely related to sepsis-associated acute brain injury. This result is in accordance with previous reports (9, 14). Age-related reconstruction of the brain tissue with senescence of astroglia results in SAE progression and neurological deficits (25). MAP was another important risk factor in predicting SAE. Magnetic resonance imaging (MRI) of patients with SAE showed cerebral ischemic lesions, indicating that a reduction in cerebral blood flow may cause SAE (26). According to the latest Surviving Sepsis Campaign guidelines, to maintain organ perfusion, fluid resuscitation and vasopressors should be introduced as early as possible to meet MAP target of greater than 65 mm Hg (27). Tissue perfusion is essential for septic patients, and the maintenance of cerebral perfusion may be critical to improving outcomes in SAE (28).

The heart rate was also another important effector in predicting occurrence for sepsis-associated acute brain injury, which is consistent with our previous study (2). Heart rate variability to predict sepsis have also been explored. Continuous heart rate variability monitoring contributes to rapid diagnosis and early intervention for severe sepsis, altering the occurrence of sepsis associated disease (29). Additionally, we found that the level of hemoglobin was associated with sepsis-associated acute brain injury. Our results suggested that low hemoglobin was found to be more likely observed in patients with sepsis-associated acute brain injury. A recent work by Wu et al. (30) suggests that lncRNA Neat1 regulates neuronal dysfunction via stabilization of hemoglobin subunit beta in SAE. Following from this, hemoglobin is an important risk factor for SAE. Finally, we also observed a significant association between length of ICU stay and sepsis-associated acute brain injury. As is well known, delirium is associated with prolonged ICU stay and hospital stay (31). Therefore, shorter ICU stay may help prevent the occurrence of sepsis-associated acute brain injury.

Alternatively, in the present study, we found that the most common complication following sepsis-associated acute brain injury was AKI with an incidence of 72.5%. Firstly, sepsis was assumed to be the leading cause of AKI in critically ill patients (32), given that up to 67% of the patients with AKI were septic in this study. Secondly, the kidney and brain closely interact in many regulation loops such as sodium and water balance or blood pressure regulation (33). After the development of sepsis-associated acute brain injury, there are two pathophysiological processes that may lead to AKI, one is the neuroendocrine pathway such as atrial natriuretic peptide secretion, the other is the inflammatory and immune pathway such as IL-6 (34).

Our study has some limitations. First, a causal relationship between the predictors and SAE cannot be established from this observational study. Second, there is not a specific diagnostic method for SAE. SAE remains a rule-out definition, which may result in a high sensitivity, but relatively low specificity. However, several factors causing impaired conscious level, including hypoglycemia, history of psychoactive drugs or alcohol, and other primary neurological disorders are excluded. Third, the assessment tool for ICU delirium is not clear in the MIMIC-III database, which is difficult in formally establishing the presence of SAE. Therefore, we described a patients-cohort with and without sepsis-associated acute brain injury. Furthermore, for GCS score were extracted before any administration of sedative drug, the use of sedative drug at ICU admission was not included in this study. Many unmeasured confounders may contribute to the impact on the prediction of sepsis-associated acute brain injury. Finally, external validation from other regions or other countries is missing, thus our results require further validation.

## Conclusion

In conclusion, we found that almost half of patients admitted to the ICU with sepsis had sepsis-associated acute brain injury. The LGBM model better identify patients with sepsis-associated acute brain injury than did other machine-learning prediction models. In addition to GCS score, glucose, age, and MAP were the three most important clinical factors to predict occurrence of sepsis-associated acute brain injury. Potentially modifiable factors associated with sepsis-associated acute brain injury at ICU admission included heart rate, hemoglobin, and length of ICU stay. These factors are likely to play a pivotal role in SAE pathophysiology, but the true causal relationship remains to be further validated.

## Data availability statement

The original contributions presented in this study are included in the article/Supplementary material, further inquiries can be directed to the corresponding author/s.

## Ethics statement

This study was an analysis of a third-party anonymized publicly available database with pre-existing institutional review board (IRB) approval. The Institutional Review Boards at the Beth Israel Deaconess Medical Center (protocol 2001-P-001699/14) and Massachusetts Institute of Technology (protocol 0403000206) have approved the data collection and the use of MIMIC-III for research purposes and granted waiver of informed consent. All methods were carried out in accordance with relevant guidelines and regulations.

## Author contributions

CG and FD analyzed the data and co-wrote the manuscript. WC and ZY collected the data. YZ prepared the figures and tables. LZ and YA were helpful for statistical analysis and interpretation of results. QP designed and revised the manuscript. All authors have reviewed the final manuscript.

## Footnotes

1
https://github.com/BboyT/SAE/tree/main

2
https://www.stata.com/

3
https://www.r-project.org/
