# Supplementary material for: Machine learning for early prediction of sepsis-associated acute brain injury
Source: Front Med (Lausanne). 2022 Oct 3;9:962027. doi: 10.3389/fmed.2022.962027 (PMC9575145; doi:10.3389/fmed.2022.962027)
Supplement: Supplementary file 1 [file Table_1.DOCX]

**TABLE S1 |** All screening variables contained missing values of less than 5%.

| **Variables** | **Missing number (%)** |  |
| --- | --- | --- |
| Gender (Male) | 0 (0) |  |
| Age | 0 (0) |  |
| Weight | 0 (0) |  |
| **Comorbidity** |  |  |
| Cardiovascular diseases | 0 (0) |  |
| Peripheral vascular diseases | 0 (0) |  |
| Hypertension | 0 (0) |  |
| Chronic pulmonary disease | 0 (0) |  |
| Diabetes | 0 (0) |  |
| AKI | 0 (0) |  |
| Obesity | 0 (0) |  |
| Liver disease | 0 (0) |  |
| Coagulopathy | 0 (0) |  |
| ARDS | 0 (0) |  |
| History of TBI | 0 (0) |  |
| History of stroke | 0 (0) |  |
| Anemia | 0 (0) |  |
| Other neurological diseases | 0 (0) |  |
| **Vital signs** |  |  |
| Mean heartrate (min^-1^) | 0 (0) |  |
| Mean arterial pressure (mmHg) | 0 (0) |  |
| Mean respiratory rate (min^-1^) | 0 (0) |  |
| Mean temperature (°C) | 124 (2.7%) |  |
| Mean SpO2 (%) | 6 (0.1%) |  |
| **Laboratory tests** |  |  |
| WBC (K/uL) | 0 (0) |  |
| Platelet (K/uL) | 64 (1.4%) |  |
| Hemoglobin (g/dL) | 1 (0.02%) |  |
| Glucose (mg/dL) | 7 (0.16%) |  |
| Potassium (K/uL) | 5 (0.11%) |  |
| Sodium (K/uL) | 2 (0.04%) |  |
| Creatinine (K/uL) | 1 (0.02%) |  |
| PCO2 (mmHg) | 0 (0) |  |
| BC (mmol/L) | 1 (0.02%) |  |
| PH | 0 (0) |  |
| Lactate (mmol/L) | 0 (0) |  |
| BE | 0 (0) |  |
| **Treatment** |  |  |
| RRT | 0 (0) |  |
| Mechanical ventilation | 0 (0) |  |
| Vasopressor | 0 (0) |  |
| **Risk scores** |  |  |
| SOFA | 0 (0) |  |
| GCS | 0 (0) |  |
| SAPSII | 0 (0) |  |
| **Infection site, n (%)** |  |  |
| Intestinal infection | 0 (0) |  |
| *(Continued)* | | |
| **TABLE S1 \|** Continued |  |  |
| **Variables** | **Missing number (%)** |  |
| Urinary infection | 0 (0) |  |
| Lung infection | 0 (0) |  |
| Catheter related | 0 (0) |  |
| Skin soft tissue | 0 (0) |  |
| Abdominal cavity | 0 (0) |  |
| **Microorganisms, n (%)** |  |  |
| Gram-positive | 0 (0) |  |
| Gram-negative | 0 (0) |  |
| Fungus | 0 (0) |  |
| Virus | 0 (0) |  |
| **Length of stay** |  |  |
| LOS in hospital (days) | 0 (0) |  |
| Length of ICU stay | 0 (0) |  |

*ARDS acute respiratory distress syndrome, AKI acute kidney injury, WBC white blood cell, BE base excess, BC bicarbonate concentration, RRT renal replacement therapy, SOFA sequential organ failure assessment, GCS Glasgow coma scale, SAPSII simplified acute physiology score*
